# Widespread Corticothalamic Connectivity Identifies the Inferior Pulvinar as a Central Node in Visual Network Architecture

**DOI:** 10.64898/2026.03.02.709198

**Published:** 2026-03-04

**Authors:** William C. Kwan, Angela Y. Fan, Andrea J Romanowski, Inaki-Carril Mundinano, Mitchell J. de Souza, James A. Bourne

**Affiliations:** 1 Australian Regenerative Medicine Institute, Monash University, Clayton, VIC 3800, Australia.; 2 National Institute of Mental Health, Bethesda, MD 20892, United States of America

## Abstract

The medial subdivision of the inferior pulvinar (PIm) has been implicated in motion processing, visuomotor integration, and residual visual function, yet a comprehensive account of its cortical inputs remains unresolved. Previous studies often relied on indirect cortical injections or tracer deposits spanning multiple pulvinar subdivisions, limiting anatomical specificity. Here, we used MRI-guided, cytoarchitectonically restricted retrograde tracer injections to selectively target PI in the common marmoset (*Callithrix jacchus*) and systematically map its cortical afferents.

Across four cases, retrogradely labeled neurons were widely distributed throughout occipital, temporal, parietal, and cingulate cortices, with a strong predominance in layer V, consistent with driver-like corticothalamic projections. Early and middle-tier visual areas (V1, V2, V3, V3A, V4, V6/DM) contributed substantial input, with labeling patterns corresponding to peripheral visual field representations. The middle temporal complex (MT, MTc, MST, FST) represented one of the densest sources of cortical projections. Prominent inputs also arose from posterior parietal regions, including LIP, MIP, VIP, AIP, and inferior parietal areas (e.g., PFG, OPt), linking PIm to visuospatial and action-related networks. Semi-quantitative analyses indicated that occipital cortex and the MT complex together accounted for approximately 60% of total cortical input, while parietal cortex contributed roughly 20%. Additional projections from retrosplenial and posterior cingulate cortices were observed.

These findings identify PIm as a central integrative node embedded within distributed visual and visuomotor networks. Rather than functioning as a restricted visual relay, PIm appears positioned to coordinate motion, spatial, and action-relevant signals within cortico-thalamo-cortical circuits supporting adaptive visually-guided behavior.

## 1. Introduction

Thalamocortical projections play a critical role in the transmission of sensory inputs from the periphery to the cortex. Within the visual sensory modality, the lateral geniculate nucleus (LGN) serves as the primary visual thalamic nucleus. Given the substantial connectivity between the retina and the primary visual cortex (V1), the body of literature that investigates thalamic control and modulation of vision is LGN-centric. Another visual thalamic nucleus of note is the pulvinar. Despite being the largest thalamic nuclei in anthropoids (Ohye, 2002), the pulvinar has attracted significantly less investigation, which could be owed to the fact that retinal afferents within the pulvinar are less prolific when compared to the LGN and even the superior colliculus within the midbrain (Kwan et al., 2019). Whilst the pulvinar is somewhat enigmatic, the immense body of work which profiled the cytoarchitecture of the pulvinar (see Baldwin and Bourne, 2020 for a comprehensive review) and mapped pulvino-cortical circuits through neural tracers (Kaas and Lyon, 2007) has provided invaluable insights into its functional role in vision.

**Figure 1:**
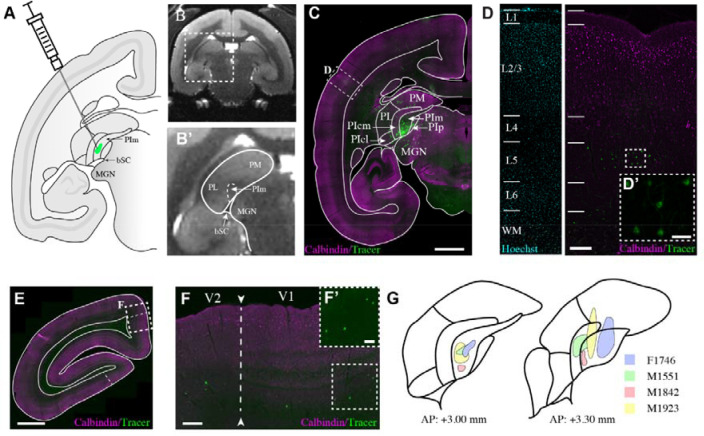
Mapping cortical inputs to the medial subdivision of the inferior pulvinar (PIm) with retrograde neural tracer injections. **A.** Schematic of the marmoset brain, the pulvinar nucleus, and PIm in coronal view. **B.** T2 MRI of the marmoset brain. **B’**. Zoomed-in image of pulvinar from **B. C.** Low-powered photomicrograph of case M1842 following tracer deposition in the temporal cortex at coordinate AP: +3.00mm. scale bar = 2mm **D.** Retrograde label is largely restricted to layer 5, as evident in **D’**. The scale bars in the low- and high-powered photomicrographs are 500 μm and 50 μm, respectively. **E.** Low-powered photomicrograph of case M1842 following tracer deposition in the occipital cortex at coordinate AP: −5.00 mm. Scale bar = 2 mm. **F.** Higher powered photomicrograph of the visual cortex as outlined in E. As evident in **F’**, V1 and V2 projections to PIm are localised in infragranular layers. Scale bar = 500 μm and 100 μm in F and F’, respectively. **G.** Summary of the location of the tracer bolus in each subject.

The pulvinar is divided into three major subdivisions: the medial (PM), lateral (PL), and inferior (PI) pulvinar. PM, the largest division, is interconnected with cortical areas spanning multiple sensory modalities and with frontal regions involved in cognitive and executive functions (Homman-Ludiye et al., 2019; Córdoba-Claros et al., 2025). In contrast, PL and PI project predominantly to visual cortical areas (Kaas and Lyon, 2007). PI can be further subdivided into four subnuclei: posterior (PIp), medial (PIm), central medial (PIcm), and central lateral (PIcl) (see Fig. 1C).

A defining feature of PI is its receipt of direct retinal input, with PIm serving as the principal locus of retinal terminations (O’Brien et al., 2001; Warner et al., 2010). PIm is distinguished by the absence of calbindin immunoreactivity and is bordered by calbindin-rich PIcm and PIp (Cusick et al., 1993; Stepniewska and Kaas, 1997). Notably, PIm contains a complete retinotopic representation of the visual field (Mundinano et al., 2019; Arafune-Mishima et al., 2020). Taken together, this evidence supports the proposal that PIm may function as a first-order visual thalamic relay analogous to the LGN (Sherman and Guillery, 1998).

PIm projects to multiple cortical areas (Kaas and Lyon, 2007), with its projection to the middle temporal area (MT) being particularly well characterized (Warner et al., 2010). MT develops in parallel with primary sensory cortices (Bourne and Rosa, 2005) and plays a central role in motion processing (Born and Bradley, 2005). The PIm–MT pathway has attracted considerable interest as a potential substrate for residual visual function following V1 lesions, commonly referred to as blindsight (Warner et al., 2010; Mundinano et al., 2018; Kinoshita et al., 2019; Fox et al., 2020; Takakuwa et al., 2021).

Beyond its proposed role in blindsight, the broader contribution of PIm to visual processing remains unclear. PIm receives input predominantly from wide-field retinal ganglion cells, with minimal contribution from midget and parasol cells that dominate LGN projections (Grünert et al., 2021). Subcortically, PIm connectivity is relatively restricted, forming connections with other retinorecipient nuclei including the parabigeminal nucleus (Diamond et al., 1992), pregeniculate, and subgeniculate nuclei (Kwan et al., 2019). In contrast, projections from the superior colliculus and LGN are sparse in PIm but dense in adjacent PIcm and PIp (Kwan et al., 2019). Electrophysiological recordings demonstrate that PIm neurons respond to moving stimuli, with a subset receiving descending input from MT exhibiting direction selectivity (Berman and Wurtz, 2011). Functional imaging further indicates responsiveness to looming stimuli (Cléry et al., 2020). Lesion studies suggest that disruption of PIm impairs visuomotor development (Mundinano et al., 2018) and visual attention to target objects (Snow et al., 2009; Wilke et al., 2010).

Because the literature concerning the functional organization of the PIm remains comparatively fragmented, we were motivated to generate a cortical connectivity map centered specifically on this subdivision of the inferior pulvinar. Establishing a detailed and anatomically precise framework of PIm afferent connectivity is a necessary first step toward formulating targeted hypotheses about its circuit-level contributions to visual and visuomotor processing. Although neuroanatomical tracing studies examining pulvinar connectivity are not unprecedented, most prior investigations have inferred PIm connectivity indirectly by placing retrograde tracers in cortical target regions and subsequently identifying labeled neurons within PIm (Kaas and Lyon, 2007; Gattass et al., 2018; Huo et al., 2019). While highly informative, this strategy does not systematically resolve the full complement of cortical inputs to PIm, nor does it permit a comprehensive evaluation of the relative strength or topographic organization of these projections. Moreover, earlier surveys that directly injected tracers into the inferior pulvinar frequently relied on relatively large tracer deposits that extended across multiple pulvinar subdivisions, thereby limiting the anatomical specificity of the resulting connectivity maps (Benevento and Rezak, 1976; Ogren and Hendrickson, 1977; Dick et al., 1991; Marion et al., 2013). Given the cytoarchitectonic and connectional heterogeneity of pulvinar subdivisions, such encroachment likely obscured subdivision-specific connectivity patterns and contributed to inconsistencies across studies.

In the present study, we therefore confined neural tracer injections predominantly to cytoarchitectonically defined PIm in the marmoset monkey (*Callithrix jacchus*), enabling a more selective and subdivision-resolved assessment of its cortical afferents. Through a systematic and extensive survey of labeled cortical neurons, we confirm several previously described PIm–cortical pathways and identify additional cortical regions that, to our knowledge, have not been reported to project to PIm. These findings refine and expand the existing model of PIm connectivity, suggesting that PIm is embedded within a broader and more integrative cortical network than previously appreciated. By establishing a reliable and reproducible methodological framework for selectively targeting PIm circuits and obtaining functional readouts, this revised network map offers a foundational resource for future studies aimed at elucidating the circuit mechanisms and behavioral significance of PIm within primate visual systems.

## 2. Materials and Methods

### Animals

Four adult New World marmoset monkeys (*Callithrix jacchus*) (1.7 – 4.1 years of age) sourced from the National Nonhuman Primate Breeding and Research Facility (Australia) were used in this study. Animals received administration of neural tracers within PIm. Following surgical procedures, the animals underwent 7 days of recovery before euthanasia. All experiments were conducted in accordance with the Australian Code of Practice for the Care and Use of Animals for Scientific Purposes, and were approved by the Monash University Animal Ethics Committee, which also monitored the health and wellbeing of the animals throughout the experiments.

### MRI Guided Microinjections

All animals in this study underwent delivery of a neural tracers under MRI guidance. Full details of preparation of animals and visualisation of target areas have been described previously (Mundinano et al., 2016). In brief, animals are anaesthetized with an intramuscular injection of alfaxalone (8mg • kg−1) combined with diazepam (3mg • kg−1) with maintenance of anaesthesia achieved with inspired isoflurane (0.5% - 2.0% in medical oxygen at 500mL • min−1). Animals were additionally medicated with atropine sulfate (50 μg • kg−1) to increase cardiac muscle contraction and reduce mucosal secretions, and with dexamethasone (40 μg • kg−1) to minimise cerebral oedema. Once an appropriate plane of anaesthesia was achieved, animals were placed in a custom-built non-ferromagnetic stereotaxic frame to facilitate T2-weighted MR Imaging. Scans were exported as Digital Imaging and Communications in Medicine (DICOM) files, and the brain images were visualized using Horos version 3.3.5 software (https://horosproject.org) to delineate the boundaries of PIm or MT. Our target area PIm received 100 nl of neural tracer at a rate of 300 nl • min^−1^ through a Hamilton Neuros Syringe (Hamilton; Cat# 65460-03) fitted with customized 33G 15° bevelled needles (Hamilton; Cat# 65461-02). At the completion of the microinjection procedure, the cranium was reconstructed and repaired with bone wax and veterinary tissue adhesive (Vetbond) as required and the scalp was sutured.

### Neural Tracers

To label descending cortical inputs to PIm, we administered a neural tracers that consisted of a [1:1] cocktail of the retrograde tracer cholera toxin subunit B (CTB) (recombinant) with Alexa Fluor 488 [10mg • ml^−1^] (Thermo Fisher Scientific, Waltham, MA; Cat# C-22841), combined with the anterograde tracer dextran amine, Alexa Fluor 488; 10,000 MW [10% w/v ] (Thermo Fisher Scientific; Cat# D22910).

### Histology and immunohistochemical tissue processing

When animals reached their endpoint (seven days post tracer delivery or at the conclusion of behavioural experimentation), they were humanely euthanized with an overdose of sodium pentobarbitone (>100mg/kg). When animals were unconscious as denoted by the absence of withdrawal and corneal reflex, animals were transcardially perfused with 10mM PBS that has been supplemented with heparin (50 IU/ml of PBS), followed by 4% paraformaldehyde in 10mM PBS. Brains were post-fixed in 4% paraformaldehyde then dehydrated in serial solutions of PBS-sucrose before being snap frozen in 2-methylbutane that has been chilled to −50°C and stored in a −80°C freezer until cryosectioning.

Brain samples were cryosectioned in the coronal plane at 50μm and stored free-floating in a cryoprotective solution (as outlined in previous work (Bourne and Rosa, 2005). Free-floating sections were labelled with the calcium-binding protein, calbindin (CB; Dilution: 1:3000; Swant cat# CB38; RRID: AB_10000340) and visualized with secondary antibody conjugated to Alexa Fluor 647 (Dilution: 1:1000; Thermo Fisher, Cat # A32795, RRID: AB_2762835) to delineate cortical and pulvinar boundaries. Sections were counterstained with Hoechst 33258 (Dilution 1:1000; Thermo Fisher, Cat # H3569; RRID: AB_2651133) to label cell nuclei and delineate cortical layers.

### Microscopy and digital image processing

Brain sections were imaged on a wide-field fluorescence microscope (Leica Thunder) using a 10x objective. Images were obtained using Leica LAS X software (Leica) at a resolution of 1.6 pixels/μm and saved in LIF format. Individual colour channels were split using FIJI (Schindelin et al., 2012) and exported as PNG. Image dimensions were scaled down to 30% of their original size with resampling to improve handling of image datasets within Adobe Illustrator. No digital adjustments were made to brightness and contrast for analysis and quantification of tracer label.

### Delineation of area boundaries and representation of tracer label

Brain section contours, boundaries, line art and digital representation of retrograde labelled cells were performed manually and drawn using Adobe Illustrator following import of fluorescently labelled microscopy images (1.6 pixels / μm). Delineation of area boundaries was undertaken by expert marmoset neuroanatomists (W.C.K, A.Y.F, I-C.M & J.A.B). We examined the laminar cytoarchitecture of Hoechst nuclear stain as well as calbindin immunolabel in the cortex for each animal and cross referenced this laminar profile with distinct anatomical landmarks such as lateral ventricle size/shape, relative position of large subcortical structures such as the SC or LGN, white matter tract size/shape.

We further cross referenced our calbindin and Hoechst label with the cortical area boundaries and nomenclature as defined in a marmoset brain stereotaxic atlas (Paxinos et al., 2012). The convergence of the labelling we observed from the calbindin and Hoechst labelling in our subjects and how areal boundaries are defined in the marmoset atlas permitted us to reach a consensus on the area boundaries within each brain slice in each of our subjects.

Following boundary delineation, cells were manually counted using the Cell Counter plugin in FIJI. For each animal, the number of PIm-projecting cells within each area was normalized to the total number of retrogradely labelled cells, yielding a semi-quantitative measure of the strength of connectivity between each cortical area and PIm.

## 3. Results

### 3.1 A primate model for characterizing PIm connectivity

To elucidate the connectivity of the medial inferior pulvinar (PIm), we mapped its projections in the common marmoset (*Callithrix jacchus*), a New World primate whose pulvinar organization closely parallels that of other primates, including humans (Münkle et al., 2000; Baldwin and Bourne, 2020), and in which the first emergence of PIm in primates. The marmoset’s small body size offers a distinct experimental advantage, enabling precise, MRI-guided subcortical microinjections (Mundinano et al., 2016) (Fig. 1A). We targeted PIm in four adult animals and delivered neural tracers via pressure injection to define its connectional architecture. Across cases, retrogradely labelled neurons were predominantly confined to layer V (Fig. 3.1C, D), revealing a striking laminar specificity. Injection sites were localized to the rostral-ventral extent of PIm (Fig. 3.1G), corresponding to peripheral visual field representations consistent with known retinotopic specializations (Mundinano et al., 2019). In subject M1842 we were able to completely restrict the tracer deposit to PIm. In subject M1551 the tracer bolus encroached on the adjacent PIcm but was still within PI. In two cases (M1923 & F1746), limited tracer backflow extended into adjacent PI subdivisions and PM. Nevertheless, all four cases were analyzed collectively and the convergent patterns observed across our dataset was cross-referenced to case M1842 to derive conclusions regarding PIm connectivity.

### 3.2 Early as well as higher-order visual cortex projects to PIm

The flow of information within the visual cortex is typically hierarchical, reflecting the progressive transformation of retinal signals as they pass through successive synaptic relays. The primary visual cortex (V1) receives the majority of retinal afferents via the thalamus and serves as the base tier in the hierarchy before signals flow onward to higher cortical areas (Felleman and Van Essen, 1991). Although strict boundaries between hierarchical “tiers” remain debated, functional and anatomical frameworks generally distinguish early visual areas (including V1 and V2), intermediate extrastriate regions within occipital cortex (e.g., V3, V3A, V4, MT-complex subdivisions), and higher-order association areas in temporal and parietal cortex that support integrative visual processing (Felleman and Van Essen, 1991; Ungerleider and Haxby, 1994; Kosslyn and Thompson, 2003).

For the purposes of this study, we defined early-tier visual cortex as V1 and V2. Middle-tier areas included remaining occipital extrastriate regions (e.g., V3 and related subdivisions), while higher-order visual areas were defined as those within temporal and posterior parietal domains implicated in advanced perceptual and visuospatial computations.

**Figure 2:**
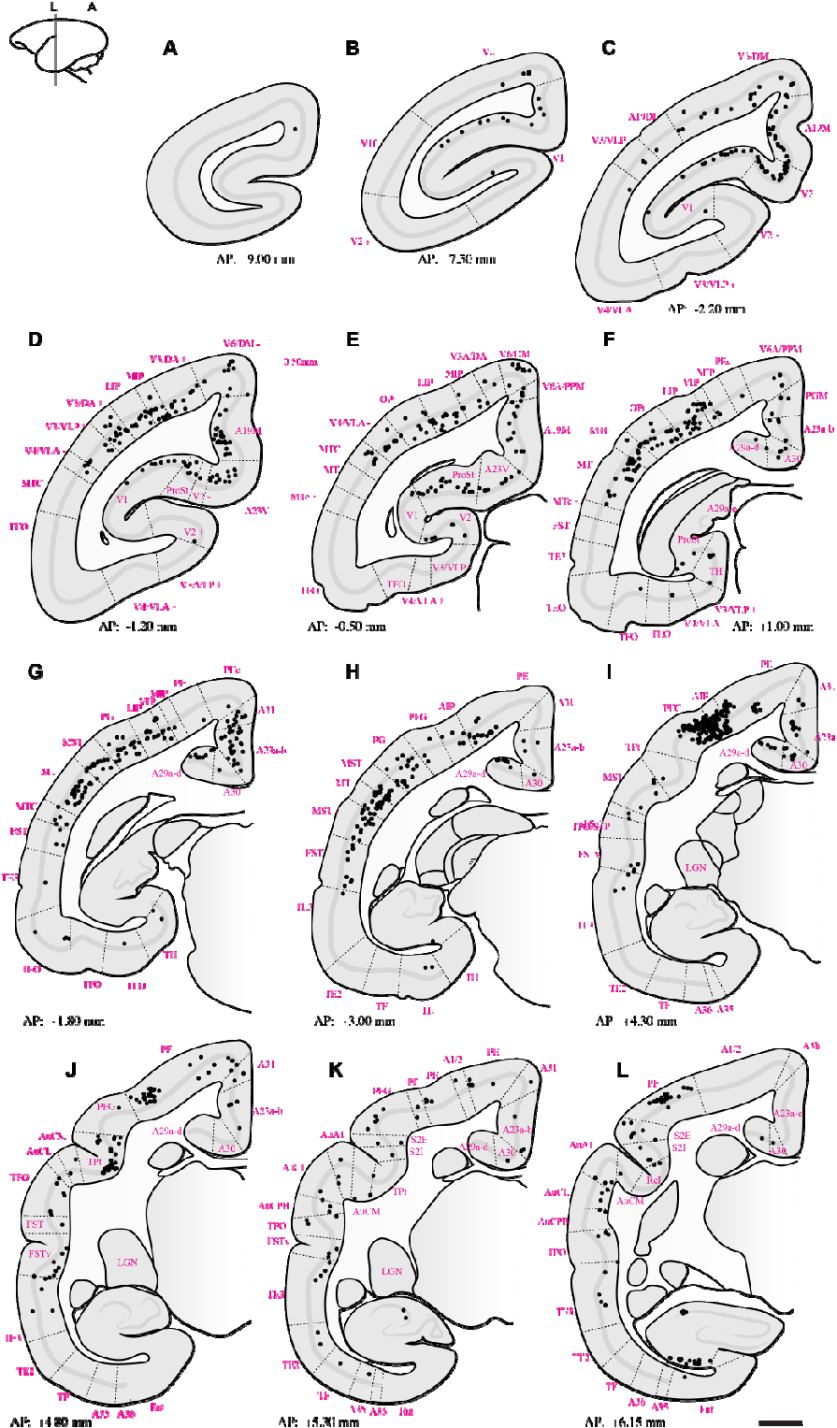
Summary of retrograde label throughout case **F1746.** Sections starting at **A.** are the most caudal, moving to the most rostral. Black dots indicate retrograde labelled cells following tracer deposition in PIm (refer to Fig 1G for tracer bolus placement). Dotted lines indicate boundaries of cortical areas. Abbreviations of cortical areas summarised in Table 1. Scale bar = 2mm.

**Figure 3:**
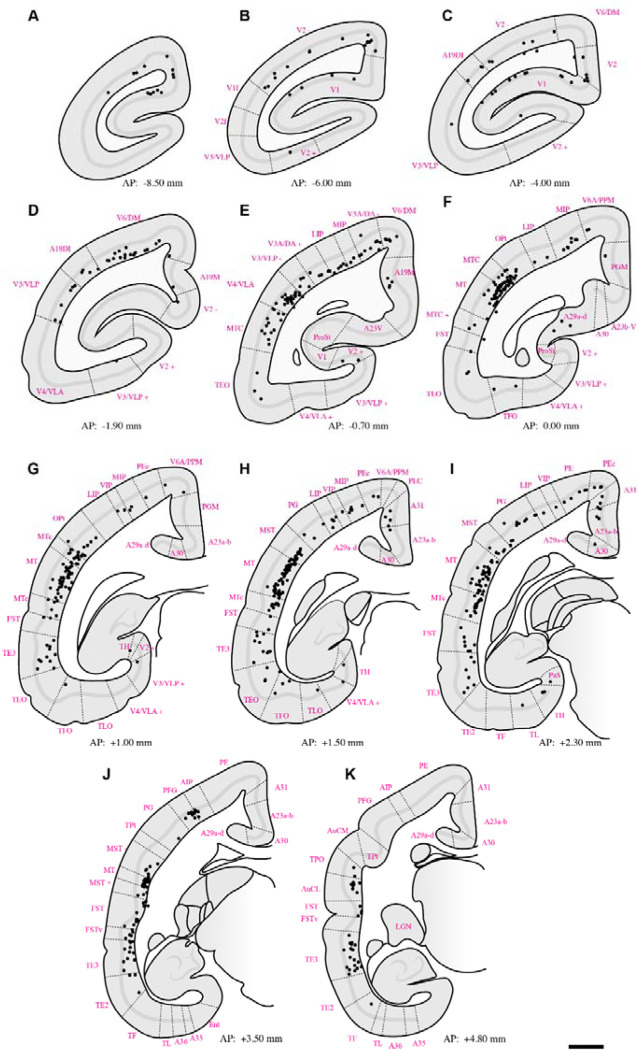
Summary of retrograde label throughout case **M1551** following deposition of tracer in PIm (refer to Fig 1G for tracer bolus placement). Dotted lines indicate boundaries of cortical areas. Abbreviations of cortical areas summarised in Table 1. Scale bar = 2mm.

**Figure 4:**
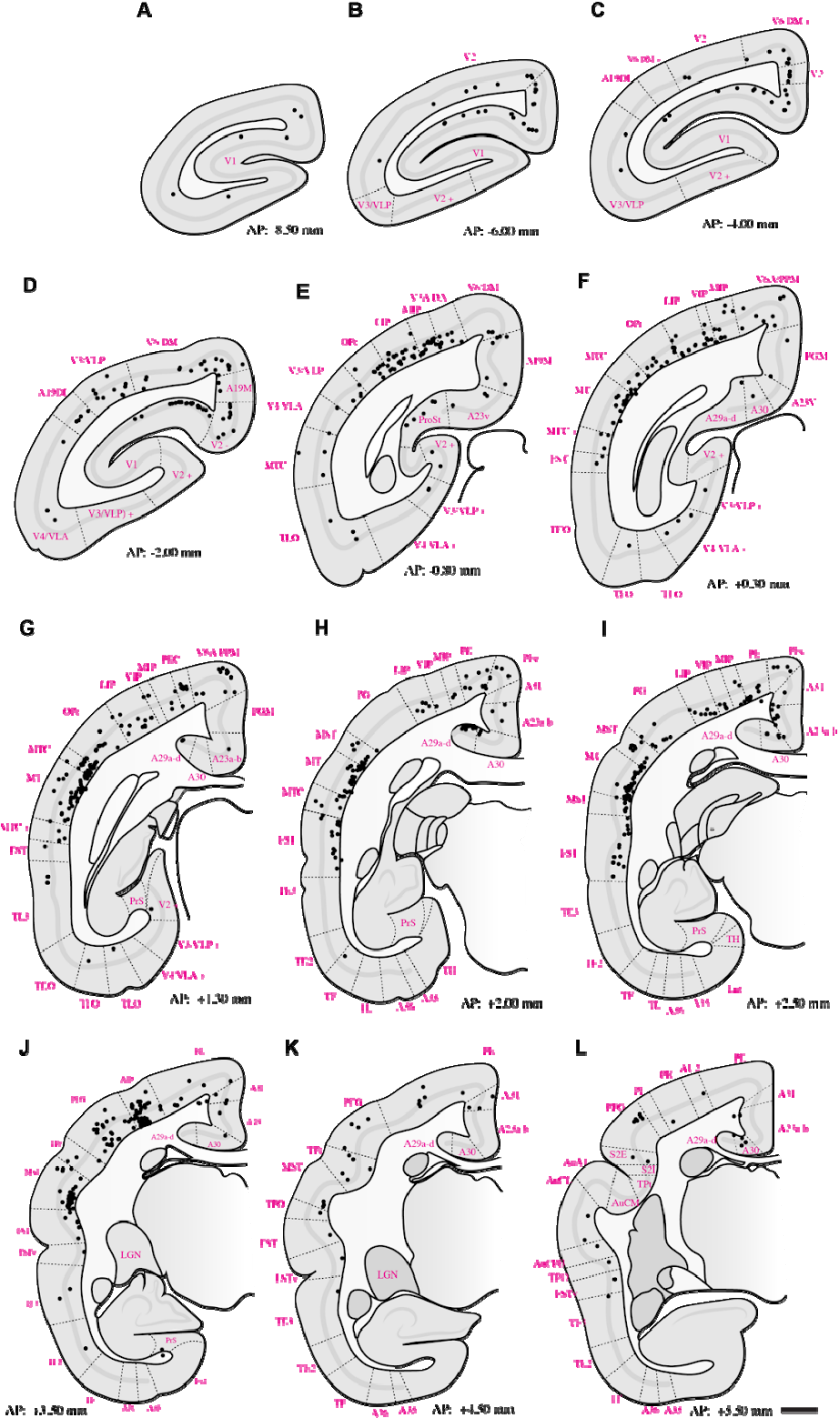
Summary of retrograde label throughout case **M1842** following deposition of tracer in PIm (refer to Fig 1G for tracer bolus placement). Dotted lines indicate boundaries of cortical areas. Abbreviations of cortical areas summarised in Table 1. Scale bar = 2mm.

**Figure 5:**
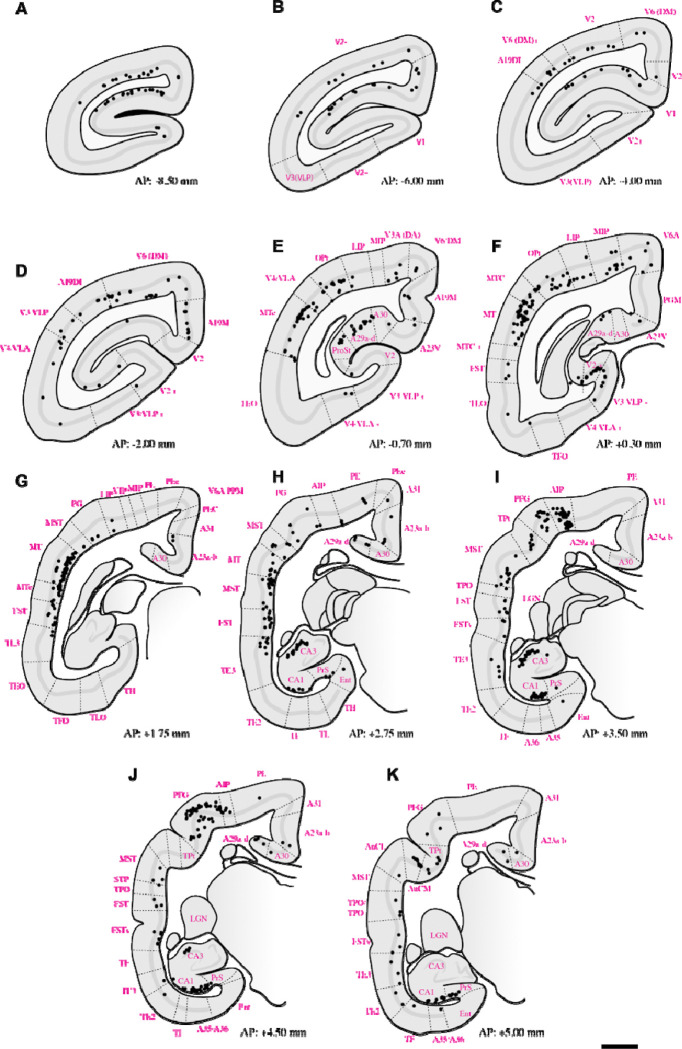
Summary of retrograde label throughout case **M1923** following deposition of tracer in PIm (refer to Fig 1G for tracer bolus placement). Dotted lines indicate boundaries of cortical areas. Abbreviations of cortical areas summarised in Table 1. Scale bar = 2mm.

Retrograde tracing revealed that PIm receives substantial input from both early and middle-tier visual areas. Labeled neurons were concentrated along the lateral and dorsal banks of occipital cortex, with a strong predominance of layer 5 projections arising from V1, V2, V3, V3A, A19DI, V4, and V6/DM (Fig. 2–5A–D). This laminar pattern is consistent with established corticothalamic projection motifs, in which layer 5 neurons provide driving input to higher-order thalamic nuclei (Sherman and Guillery, 2002), suggesting that PIm is embedded within a distributed occipital network spanning multiple hierarchical stages of visual processing.

Previous investigations of pulvinar afferent connectivity in marmoset and macaque revealed the existence of projections from early visual areas within PI (Ogren and Hendrickson, 1977; Kennedy and Bullier, 1985; Dick et al., 1991; Fitzgibbon et al., 2007; Baldwin et al., 2012) (Ogren and Hendrickson, 1976; Kennedy and Bullier, 1985; Dick et al., 1991; FitzGibbon et al., 2007; Baldwin et al., 2012). These early expeditions, which mapped visual cortical efferents in the pulvinar, only described which major subdivision received the cortical signals (e.g. the inferior pulvinar as a whole). Later studies, which examined PI connectivity, utilized calbindin immunohistochemistry to delineate PI into the four canonically described subdivisions and showed projections from early striate areas to PI primarily terminated in the subdivisions that flank PIm, i.e. PIcm, PIcl, and PL (Cusick et al., 1993; Kaas and Lyon, 2007; Gattass et al., 2018). Whilst our results of V1/V2 projections to PIm have not been widely reported, they agree with a previous study which observed V1 neurons from peripheral visuotopic representations projecting to PIm (Gutierrez and Cusick, 1997). The foveal visual field in PIm is predominantly represented by its ‘dorsomedial tail’, whilst the ventral ‘base’ of PIm, which is where we deposited tracer in all four cases, is represented by the periphery (Mundinano et al., 2019). The V1/V2 retrograde label we observed in the calcarine sulcus in more rostral portions of V1 (Fig. 2–5: A–C) corresponds to the peripheral visual field (Fritsches and Rosa, 1996) and aligns with our placement of neural tracer. We were unable to deposit tracer into the foveal representation of PIm in this study. As such, we couldn’t definitively exclude the existence of foveal connectivity between PIm and the early visual cortex. However, prior work indicates that pulvinar connectivity associated with foveal representations is minimal or absent (Kaas and Lyon, 2007). On this basis, we infer that the visual input PIm receives from the early visual cortex is preferentially weighted toward peripheral visual field representations.

The pattern of labeling observed in higher-order visual cortical areas, including V3, V3A, A19DI, and V6/DM (Fig. 2–5: C–D), is consistent with previously reported corticocortical connectivity patterns described in both New World and Old World primates (Asanuma et al., 1985; Dick et al., 1991; Yeterian and Pandya, 1997; Kaas and Lyon, 2007). These earlier tract-tracing studies demonstrated robust projections between extrastriate visual areas and posterior parietal regions, supporting the interpretation that the present labeling reflects homologous higher-order visual pathways.

To our knowledge, direct projections from V4/VLA to PIm have not previously been reported in the marmoset. In contrast, anatomical connectivity between V4 and subdivisions of the inferior pulvinar, including PIm, is well established in the macaque based on retrograde and anterograde tracer studies (Gattass et al., 2014). The present findings therefore extend existing data by identifying a comparable projection in the marmoset, suggesting that this thalamocortical network may be more evolutionarily conserved across primate lineages than previously recognized.

From regions occupying the medial surface of the extrastriate cortex and the calcarine sulcus, we observed retrogradely labelled neurons in areas A19M, A23V, and prostriata (ProSt) following retrograde tracer injections in PIm (Fig. 2–5: D, E). Although pulvinar connectivity with these areas has not previously been reported in the marmoset, a study in the macaque demonstrated axonal terminal labelling in PIm following broad anterograde tracer injections in medial extrastriate cortex (Yeterian and Pandya, 1997). Functional investigations have revealed that these areas are preferentially involved in processing visual motion perceived from the far peripheral visual field (Palmer and Rosa, 2006; Yu et al., 2012; Mikellidou et al., 2017). Consistent with this functional specialization, retrograde labelling was observed in A19M and ProSt in all four cases, and in A23V in three cases. This pattern may reflect tracer uptake within the peripheral representation in ventral PIm.

### 3.3 Projections from the temporal and parietal cortices

As visual processing evolved in nonhuman primates and humans, the computations underlying visual perception and visually guided behaviour became increasingly complex, accompanied by an expansion of extrastriate cortex into temporal and parietal domains. Subcortical circuits play a critical role in mediating information transfer between major cortical lobes, with the pulvinar serving as a key hub for regulating communication across distributed visual cortical networks (Saalmann et al., 2012). Awake functional MRI studies in nonhuman primates demonstrate that visual stimulation engages widespread pulvinocortical networks, with activation patterns varying systematically according to the nature of the visual stimulus presented (Cléry et al., 2020; Guedj and Vuilleumier, 2023). Given the proposed integrative role of the pulvinar in large-scale cortical communication, we sought to determine whether tracer labelling in our cases similarly revealed extensive connectivity with parietal and temporal cortical regions.

Extensive retrograde labelling was observed in the temporal cortex (Fig. 2–5: E–I), with area MT exhibiting the densest cortical projections to PIm. Substantial projections to PIm were also identified from MT satellite regions, including MTc, MST, and FST. The distribution of retrograde labelling within these motion-related temporal areas is consistent with previous tract-tracing studies in both Old and New World primates (Boussaoud et al., 1992; Palmer and Rosa, 2006; Kaas and Lyon, 2007).

In contrast, projections from inferior temporal areas TE3, TE2, and TEO were generally sparse. Except for subject M1551 (Fig. 3J, K), retrograde labelling in these regions was limited, with modest labelling observed in subjects F1746 (Fig. 2J, K) and M1923 (Fig. 3.5J, K), and near absence of label in M1842 (Fig. 4J–L). Although the distribution of projections within inferior temporal cortex varied across subjects, previous studies involving broad tracer injections in TE and TEO in the macaque have demonstrated projections from inferior temporal cortex to PIm (Baleydier and Morel, 1992; Baizer et al., 1993; Webster et al., 1993).

Within the parietal cortex, retrograde labelling was observed in regions surrounding the intraparietal sulcus, including AIP, MIP, LIP, and VIP, as well as in superior parietal areas PE and PEc, inferior parietal areas PF, PFG, and PG, and the occipitoparietal transitional area (OPt). Across subjects, the distribution of PIm-projecting neurons in MIP, LIP, and VIP was consistent, forming a mediolaterally distributed band of retrogradely labelled cells (Fig. 2–5: E–G). In three of four cases (Fig. 2I; Fig. 4J; Fig. 5I), AIP exhibited comparatively denser retrograde labelling than other intraparietal areas.

The presence of direct projections from LIP to PIm is consistent with findings in the macaque (Giolli et al., 2001). To our knowledge, this is the first report demonstrating connectivity between PIm and VIP, MIP, and AIP in the marmoset. Previous tract-tracing studies in Old World macaques have generally reported projections from intraparietal regions to the anterior or medial pulvinar nuclei (Baizer et al., 1993; Padberg and Krubitzer, 2006; Cappe et al., 2007; Prevosto et al., 2009).

Projections to PIm from inferior parietal areas were generally more extensive than those from superior parietal regions across all four cases (Fig. 2–5E–K). Within the inferior parietal cortex, retrograde labelling was consistently denser in OPt and PFG compared to PF and PG. Across subjects, the MT complex—comprising MT, MTc, and MST—typically exhibited the densest retrograde labelling overall (Fig. 2–5F–H). An exception was observed in case F1746, in which more rostral representations within the superior posterior parietal cortex (V6A/PPM, PEc, and PE) also showed substantial projections to PIm (Fig. 2I,J). Sparse retrograde labelling in PEc was observed in three of four cases (Fig. 2G; Fig. 3–4I).

Previous studies examining posterior parietal projections to the pulvinar have reported variable findings. Several investigations have identified the medial pulvinar (PM) as the principal recipient of posterior parietal inputs, with minimal contribution from PI subdivisions (Yeterian and Pandya, 1985; Baleydier and Mauguière, 1987; Baleydier and Morel, 1992; Impieri et al., 2018). In contrast, other studies have reported projections from parietal cortex to PIm (Kasdon and Jacobson, 1978; Brysch et al., 1990; Baizer et al., 1993).

Within the posterior parietal cortex (PPC), retrograde labelling was not uniformly distributed, suggesting that projections to PIm may arise from specific topographic subdivisions. A recent tract-tracing study in the prosimian galago reported that projections to PI originate predominantly from the dorsal-caudal PPC (Wang et al., 2023), a region implicated in visually-guided reaching behaviours. Notably, this subdivision receives input from peripheral representations of extrastriate visual cortex (Stepniewska et al., 2015). In humans, parietal lesions have been associated with impairments in reaching toward targets in the peripheral visual field, with deficits reduced following foveation (Passarelli et al., 2021). Together, these findings provide a comparative framework for interpreting the pattern of parietal projections to PIm observed in the present study.

### 3.4 Projections from other domains

Across all cases, retrograde labelling was observed in the retrosplenial cortex (areas A29 and A30) and posterior cingulate regions (A23a–b, A31, and PGM). In addition, retrogradely labelled neurons were identified in hippocampal subfields CA1 and CA3 in two cases (F1746 and M1923) which had tracer spread extended into ventral portions of PM.

Previous tract-tracing studies have primarily reported connectivity between retrosplenial, posterior cingulate cortex or hippocampus with the medial (PM) or lateral (PL) subdivisions of the pulvinar, with no clear evidence of projections to PI subdivisions (Baleydier and Mauguiere, 1985; Leichnetz, 2001; Shibata and Yukie, 2003; Buckwalter et al., 2008; Gamberini et al., 2020; Togo et al., 2025). The densest retrosplenial labelling was observed in subjects F1746 (Fig. 2F–I) and M1923 (Fig. 5E,F), the cases tracer spread into medial pulvinar. However, in case M1842, where tracer deposition was confined to PIm, whilst CA1 and CA3 label was absent, substantial retrograde labelling was also present in posterior cingulate and retrosplenial cortices (Fig. 4H,I).

### 3.5 Semi-quantitative assessment of cortical connectivity with PIm

**Figure 6:**
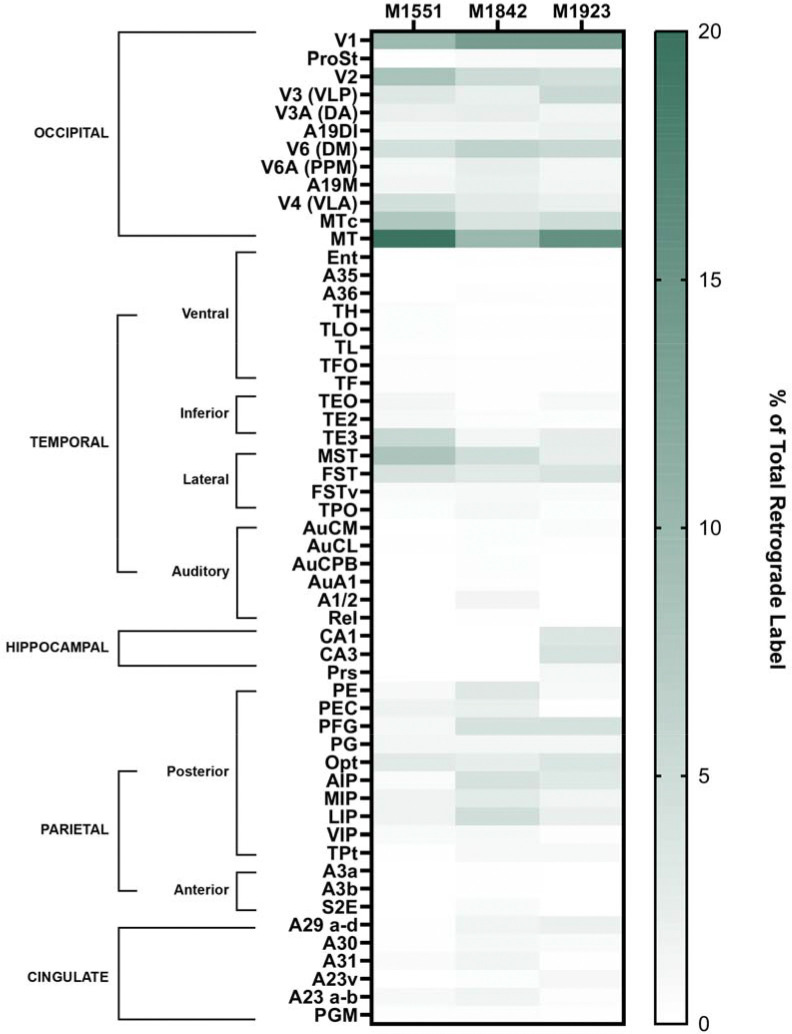
Semi-quantitative assessment of cortical contributions to PIm, calculated as the number of labeled cells in each area divided by the total number of labeled cells quantified within each subject. Areas are grouped by major regions and subregions according to the marmoset brain atlas (Paxinos et al., 2012; Córdoba-Claros et al., 2025). See Table 1 for area abbreviations.

To assess the relative strength of cortical inputs to PIm, we quantified the number of retrogradely labelled neurons in each cortical area as a proportion of the total labelled cells observed per subject (Fig. 6). Consistent with previous reports indicating that PI subdivisions are predominantly associated with visual processing, inputs from early visual areas in the occipital lobe and the MT complex in the temporal lobe accounted for nearly 60% of total retrograde labelling. V1 and MT which act as the primary early maturing nodes of the visual cortex (Bourne, 2010) together contributed approximately 25% of all cortical inputs to PIm.

The parietal cortex represented the second largest source of cortical input, contributing close to 20% of total retrograde label. In contrast to the occipital domain, where V1 and MT showed markedly greater labelling than other visual areas, the distribution of labelled neurons within the parietal cortex was comparatively uniform. Areas PFG, OPt, and LIP each accounted for approximately 3% of total labelled cells.

Retrograde labelling was also consistently observed within cingulate regions across subjects, contributing approximately 4% of the total cortical input. Contributions from the temporal cortex were more selective; while most inferior temporal areas showed limited labelling, TE3, MST, and FST together comprised over 10% of total cortical projections to PIm. In two of four cases, retrogradely labelled neurons were additionally identified within the hippocampal formation.

## 4. DISCUSSION

Our goal in undertaking this study was to reconcile a fragmented literature and move the field toward a clearer consensus on corticopulvinar circuits centered on the medial subdivision of the inferior pulvinar (PIm). Much of what we currently understand about cortical–pulvinar connectivity derives from studies in Old World monkeys, where key cortical territories are buried deep within sulci and can be challenging to access comprehensively. As the pattern of cortical inputs to a thalamic nucleus provides critical clues to its functional identity, a systematic and targeted mapping of cortical projections to PIm was needed. By placing retrograde tracers directly within PIm, we aimed to generate a more complete cortical–PIm connectivity map and, in doing so, reveal functional contributions that may have remained underappreciated

Our retrograde tracer injections into the peripheral PIm identified a distributed constellation of cortical areas projecting to this thalamic region. Considering the mosaic organization of primate visual cortex, where distinct areas exhibit eccentricity biases, some preferentially representing the fovea (Rosa and Tweedale, 2000), while others emphasize peripheral visual space (Palmer and Rosa, 2006), these findings help clarify which cortical domains preferentially engage the peripheral sector of PIm. This distinction is particularly relevant given the disproportionate role of foveal processing in primate vision. An important unresolved question is whether the foveal portion of PIm (the ‘calbindin-negative tail’) shares a similar connectional architecture or instead participates in a distinct corticopulvinar network. Prior work suggests that direct input from foveal representations of early visual cortex may be sparse, as tracer injections restricted to the marmoset V1 operculum (0–5° eccentricity) did not label PIm (Kaas and Lyon, 2007). However, anterograde tracer placements near the V1–V2 border have revealed axonal terminations within PIm (Gutierrez and Cusick, 1997), leaving open the possibility that specialized corticopulvinar circuits selectively link foveal cortex with this thalamic territory.

Several limitations should be noted. The first is that only one case had tracer restricted entirely in PIm. Most notably, in two cases, some tracer encroached on the ventral portion of PM. In these two cases alone, we observed projections from the hippocampus, and in the case where PIcm was also injected, this subject had a higher proportion of cortical inputs from the inferior temporal domain. Nevertheless, after normalizing the total retrograde label in each subject, that it was clear the distribution of cortical afferents across all subjects converged to show a similar spatial profile (Fig 6). The other limitation worth highlighting is that the extensive cortical labeling observed after PIm injections precluded a fully reciprocal strategy in which tracers would be placed in every retrogradely labeled cortical area. Instead, we interpreted our findings in the context of the substantial corticopulvinar literature in monkeys, where tracers were typically administered in defined cortical fields. Our analysis also prioritized occipital and visual cortices; technical limitations during cryosectioning limited recovery of the frontal pole, and thus, potential rostral cortical inputs to PIm were not systematically examined. Frontal projections to the pulvinar are generally reported to converge within the medial pulvinar (Brysch et al., 1990; Romanski et al., 1997; Roberts et al., 2007; Homman-Ludiye et al., 2019; Córdoba-Claros et al., 2025), although sparse projections from PI and PIm to areas 8A and 46v have been described (Romanski et al., 1997; Córdoba-Claros et al., 2025). The opportunity to reveal additional frontal inputs to PIm appears unlikely, given the visual specialization of PI, although a truly comprehensive account of corticopulvinar organization will ultimately require direct examination of rostral cortices. Species differences also merit consideration. Most pulvinocortical tracing studies have been conducted in macaques, and discrepancies with prior reports (e.g., parietal or retrosplenial connectivity with PIm) may reflect marmoset-specific organization. Whilst species-specific attributes will always be inevitable, methods such as consensus mapping, a technique that unifies species-specific anatomical atlases using a common parcellation scheme, offer an elegant solution for directly interrogating commonalities and differences between primate species (Magrou et al., 2026). Supporting this possibility, functional imaging in marmosets demonstrated activation of PI and PM subdivisions, along with occipital, parietal, temporal, inferior temporal, and retrosplenial cortices, during looming stimulation (Cléry et al., 2020), although fMRI cannot resolve specific corticopulvinar circuits at the PI subdivision level. As the marmoset model gains prominence for its lissencephalic cortex (Solomon and Rosa, 2014) and its suitability for social neuroscience (Miller et al., 2016; Scott and Bourne, 2022), accurate species-specific connectional maps are increasingly important. Future tracer studies targeting underexplored regions, including medial extrastriate and posterior cingulate cortices, will be necessary to refine the corticopulvinar framework proposed here.

Collectively, our findings position PIm as a central integrative node within the primate visual system, receiving convergent input from occipital, temporal, and parietal cortices. This extensive connectivity reinforces the emerging view of the pulvinar as an active regulator of cortical communication, dynamically shaping excitatory and inhibitory interactions across distributed networks (Kohn et al., 2020). The distribution of retrograde labeling indicates that PIm incorporates signals from multiple tiers of the visual hierarchy, including the middle temporal complex, a core substrate for motion processing (Rosa and Elston, 1998). Importantly, projections from intraparietal regions involved in reach and grasp (Vesia and Crawford, 2012), superior posterior parietal areas supporting visuomotor guidance and body-in-space awareness (Passarelli et al., 2021), and inferior parietal territories implicated in extrapersonal spatial processing (Kravitz et al., 2011) extend PIm’s function beyond perception and into action-oriented visual domains.

Viewed in this light, PIm is not merely a relay within the visual thalamus but a nexus where perceptual, spatial, and visuomotor signals converge, but a flexible and plastic node that could enable the coordination of sensory inputs with behavioral demands. Although retrosplenial and posterior cingulate cortices, which contribute to navigation and spatial memory (Vann et al., 2009; Leech and Smallwood, 2019), remain less clearly linked to PIm in our data, an even broader sensory integration repertoire remains a possibility. Taken together, the connectional architecture described here supports a model in which PIm participates in orchestrating multidimensional visual representations, aligning motion, space, and action within a unified thalamocortical framework.

**Figure 7:**
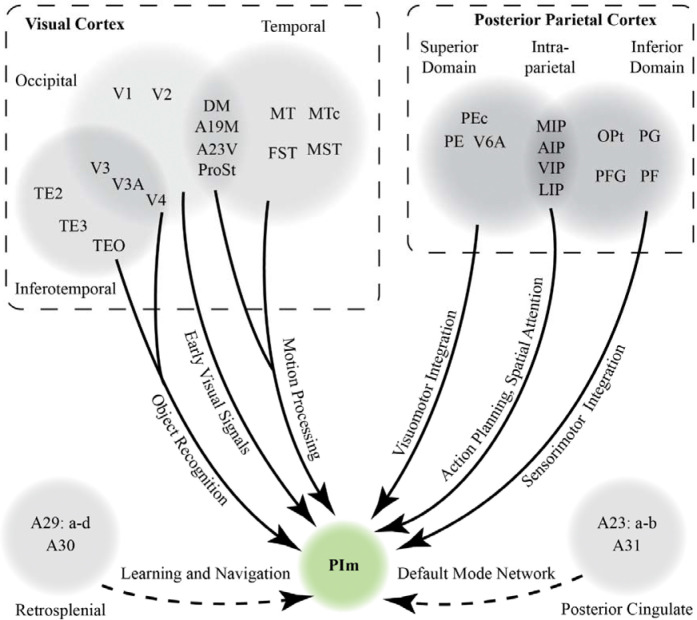
Summary of the retrograde tracing results in this study and our proposed network of PIm cortical connectivity. Solid lines denotes the functional domains PIm receives input from which were observed in this study and supported by other previous literature in the nonhuman primate. Dashed lines represent projections from areas which are not definitive due to tracer encroaching in adjacent areas and the lack of previous literature reporting afferents from that domain.

Existing circuit-level evidence further supports an integrative role for PIm. This subdivision receives input from diverse classes of wide-field retinal ganglion cells that are sensitive to visual motion, looming stimuli, and signals relevant for visual pursuit (Puller et al., 2015; Grünert et al., 2021; Kim et al., 2022), positioning it to access rapid, behaviorally salient visual information. PIm has also been implicated in the development of goal-directed reach and grasp behaviours (Mundinano et al., 2018) and in filtering visual distractors (Snow et al., 2009), highlighting its contribution to action selection and attentional control. Considered alongside the extensive cortical inputs identified here, from motion-sensitive visual areas and parietal visuomotor regions, PIm appears strategically situated as the conduit for perception and action. Rather than functioning as a passive relay, PIm may serve as an early thalamic gatekeeper that primes and stabilizes visuomotor networks, aligning salient visual signals with the coordinated execution of hand–eye behaviors such as reach and grasp (Fig. 7).

Taken together, our findings support a model in which the inferior pulvinar, particularly the PIm subdivision, serves as a principal hub for coordinating corticocortical interactions. The cortico-thalamo-cortical loops enable the observer to rapidly interpret and anticipate appropriate motor responses to dynamic visual input, consistent with the action-oriented functions ascribed to the dorsal stream (Goodale and Milner, 1992). Beyond visuomotor integration, connectivity between PI and the amygdala, a pathway implicated in processing fearful and behaviorally salient stimuli (McFadyen et al., 2019) raises the possibility that the inferior pulvinar operates as an early responder to biologically relevant sensory events (Soares et al., 2017). In natural environments, visual stimuli vary widely in emotional and behavioral significance, often demanding responses that extend beyond stereotyped reach-and-grasp actions to include vocalization, defensive reactions, or higher-order cognitive strategies. Although PI lacks strong direct projections to frontal cortex, its influence may nevertheless propagate to executive networks through short polysynaptic routes. Notably, the PM nucleus projects robustly to frontal cognitive centers, and overlapping temporal (e.g., TE3, TE2) and parietal (e.g., PFG, PG) territories interface with both PI and PM. These shared circuit motifs suggest that PI and PM cooperate via distributed temporal–parietal loops to regulate information flow between early visual areas and higher-order cognitive systems. Such a framework resonates with the global neuronal workspace model (Cortes et al., 2024), in whichu ascending pulvinar projections amplify and sustain cortical signals, preventing their attenuation before they reach distributed cognitive networks. Testing these hypotheses will require technically demanding circuit-level manipulations, yet emerging genetic technologies, coupled with increasingly sophisticated quantification of naturalistic primate behavior, now make such investigations feasible. By establishing a comprehensive anatomical framework of the PIm network, the present study lays critical groundwork for future efforts to decipher how the pulvinar coordinates brain-wide communication, transforming transient sensory signals into coherent, adaptive behavior.

## Figures and Tables

**Table 1:** Abbreviations of cortical areas in the marmoset monkey. Nomenclature used follows the marmoset brain stereotaxic atlas (Paxinos et al., 2012)

Abbreviation	Full Name
A19DI	Area 19 of cortex, dorsointermediate part
A19M	Area 19 of cortex, medial part
A23a-b	Area 23a-b of cortex
A23V	Area 23 of cortex, ventral part
A29a-d	Area 29a-d of cortex
A30	Area 30 of cortex
A31	Area 31 of cortex
A35	Area 35 of cortex
A36	Area 36 of cortex
AuA1	Auditory cortex, primary area
AuCL	Auditory cortex, caudolateral area
AuCM	Auditory cortex, caudomedial area
AuCPB	Auditory cortex, caudal parabelt area
AuML	Auditory cortex, middle lateral area
CA1	Hippocampus field CA1
FST	Fundus of superior temporal sulcus area of cortex
LIP	Lateral intraparietal area of the cortex
MIP	Medial intraparietal area of the cortex
MST	Middle superior temporal area of cortex
MT (V5)	Middle temporal area/visual area 5
MTc	Middle Temporal Crescent
OPt	Occipito-parietal transitional area of the cortex
PE	Parietal area PE
PEC	Parietal area PE, caudal part
PF	Parietal area PF
PFG	Parietal area PFG
PG	Parietal PG, medial
FSTv (PGa-Ipa)	Fundus of superior temporal ventral area (Parietal Areas PGa and IPa)
PGM	Parietal area PG, medial part
ProSt	Prostriate area
TE2	Temporal area TE2 (inferior temporal cortex)
TE3	Temporal area TE3 (inferior temporal cortex)
TEO	Temporal area TE, occipital part
TF	Temporal area TF
TFO	Temporal area TF, occipital part
TH	Temporal area TH
TL	Temporal area TL
TLO	Temporal area TL, occipital part
TPt	Temporoparietal transitional area
V1	Primary visual cortex
V2	Visual area 2
V3/VLP	Visual area 3/ventrolateral posterior area
V3A/DA	Visual area 3A/dorsoanterior area
V4/VLA	Visual area 4/ventrolateral anterior area
V6/DM	Visual area 6/dorsomedial area
V6A/PPM	Visual area 6A/posterior parietal medial area)
VIP	Ventral intraparietal area of cortex
